# Purification and Characterization of a DegP-Type Protease from the Marine Bacterium *Cobetia amphilecti* KMM 296

**DOI:** 10.3390/microorganisms11071852

**Published:** 2023-07-21

**Authors:** Yulia Noskova, Oksana Son, Liudmila Tekutyeva, Larissa Balabanova

**Affiliations:** 1 Laboratory of Marine Biochemistry, G.B. Elyakov Pacific Institute of Bioorganic Chemistry, Far Eastern Branch, Russian Academy of Sciences, Prospect 100-Letya Vladivostoka 152, 690022 Vladivostok, Russia; 2 Advanced Engineering School, Institute of Biotechnology, Bioengineering and Food Systems, Far Eastern Federal University, 10 Ajax Bay, Russky Island, 690922 Vladivostok, Russia; oksana_son@bk.ru (O.S.); tekuteva.la@dvfu.ru (L.T.)

**Keywords:** marine bacterium, *Cobetia amphilecti*, DegP-type periplasmic serine endoprotease, recombinant protein, heterologous expression, proteolytic activity, physical and chemical properties

## Abstract

A new member of the DegP-type periplasmic serine endoproteases of the S1C family from the marine bacterium *Cobetia amphilecti* KMM 296 (CamSP) was expressed in *Escherichia coli* cells. The calculated molecular weight, number of amino acids, and isoelectric point (pI) of the mature protein CamSP are 69.957 kDa, 666, and 4.84, respectively. The proteolytic activity of the purified recombinant protease CamSP was 2369.4 and 1550.9 U/mg with the use of 1% bovine serum albumin (BSA) and casein as the substrates, respectively. The enzyme CamSP exhibited maximum activity at pH 6.0–6.2, while it was stable over a wide pH range from 5.8 to 8.5. The optimal temperature for the CamSP protease activity was 50 °C. The enzyme required NaCl or KCl at concentrations of 0.3 and 0.5 M, respectively, for its maximum activity. The Michaelis constant (*K*_m_) and V_max_ for BSA were determined to be 41.7 µg/mL and 0.036 µg/mL min^−1^, respectively. The metal ions Zn^2+^, Cu^2+^, Mn^2+^, Li^2+^, Mg^2+^, and Ca^2+^ slightly activated CamSP, while the addition of CoCl_2_ to the incubation mixture resulted in a twofold increase in its protease activity. Ethanol, isopropanol, glycerol, and Triton-X-100 increased the activity of CamSP from two- to four-times. The protease CamSP effectively degraded the wheat flour proteins but had no proteolytic activity towards soybean, corn, and the synthetic substrates, α-benzoyl-Arg-p-nitroanilide (BAPNA) and N-Succinyl-L-alanyl-L-alanyl-L-prolyl-L-phenylalanine 4-nitroanilide (SAPNA).

## 1. Introduction

Proteases (peptidases, proteolytic enzymes) belong to a large group of hydrolases with different structures and biological functions that catalyze the cleavage of peptide bonds in proteins. They are widely distributed in nature, both in microorganisms and in animal and plant cells [1]. Proteases control the activation, synthesis, and turnover of proteins for various physiological processes in cells [2,3,4]. Depending on their localization in the cell, and the arrangement of amino acid (aa) residues in the active center and mechanism of action, proteases are classified into exo- or endoproteases belonging to different families, such as serine, aspartic, cysteine, and many others [5,6,7].

The representatives of each structural family are described in detail in the peptidase database MEROPS (http://merops.sanger.ac.uk/, accessed on 20 April 2023). Each peptidase is assigned to a family based on statistically significant similarities in the amino acid sequence, and families that are thought to be homologous grouped together in a Clan by indexes [5]. The S1C subfamily of DegP-type periplasmic serine endoproteases is a group of proteins that have a chymotrypsin-like fold, with a single domain, and play a dual role in bacterial cells [5,8]. They act as chaperones at low temperatures, helping to fold and stabilize other proteins, but switch to the peptidase function at high temperatures, degrading unfolded or misfolded proteins that accumulate in the periplasm following heat shock or other stress conditions [9,10,11,12]. These enzymes are essential for bacterial survival and virulence at high temperatures (above 42 °C), which generate misfolded proteins and participate in the biogenesis of partially folded outer-membrane proteins (OMPs). Therefore, the bacterial DegP may be used as the target in both drug discovery and infectious disease treatment [13,14]. In mammals, the dysfunction of protease HtrA2, which is structurally similar to the bacterial DegP-type proteases, is associated with cancer and Alzheimer’s disease [8,15]. HtrA/DegP/Q-type enzymes are known to be allosterically regulated and have the catalytic triad of His-Asp-Ser and two PDZ domains that mediate substrate recognition and oligomerization, with a preference for cleaving peptide bonds between hydrophobic residues, such as Val-Val or Ile-Xaa [9,11,12,13,14,16]. Allosteric effects may be observed between the individual domains of monomeric proteins DegP or between the protomers of the oligomeric protein complexes. Moreover, allosteric switching between their active and inactive conformations is frequently accompanied by changes in their oligomeric states (up to 24-mers) [10].

Proteases have been isolated and characterized from various sources, but microorganisms are the most preferred source for industrial purposes due to their biochemical diversity, rapid growth in a limited space, and a simple nutrition medium, as well as established genetic methods [17,18]. Bacterial proteases are easily produced in large quantities and are most often thermostable and active over a wide pH range, making them available for industrial use. The enzymes isolated from the microorganisms *Bacillus* sp. [19,20,21,22], *Aspergillus* sp. [23], and some other bacteria [24] are among the proteases used in various industries, such as leather, textile, pharmaceutical, detergent, and food, including baking. Acidic proteases are obtained from fungi, while neutral proteinases are mainly of plant origin. The isolation of both acidic and neutral proteases from fungi and plants is relatively laborious and uneconomical, while alkaline proteases obtained from different types of bacteria are in great demand in industry [17].

Efforts are currently underway to find and isolate new bacteria from poorly understood habitats that can produce enzymes with unique properties suitable for industrial applications [25]. The World Ocean can become such a unique source of producers of new proteases. Much of the deep-sea environment is subjected to high pressure, high salt concentration, and low temperature, and the organisms living in these conditions have adapted to them. Great interest in the enzymes isolated from marine microorganisms is associated with their activity and stability under extreme conditions due to the habitat of these bacteria [26].

The present study presents data on the recombinant production and purification of the new neutral DegP-type protease CamSP from the marine bacterium *C. amphilecti* KMM 296 (Collection of Marine Microorganisms, G.B. Elyakov Pacific Institute of Bioorganic Chemistry, Far Eastern Branch, the Russian Academy of Sciences (PIBOC FEB RAS)), isolated from the coelomic fluid of the mussel *Crenomytilus grayanus*, and the physical–chemical and catalytic properties of the enzyme are described.

## 2. Materials and Methods

### 2.1. Reagents

High-purity-grade reagents from Merck (Munich, Germany), Sigma (OOO Sigma-Aldrich Rus, Moscow, Russia), and Helicon (Moscow, Russia) were used. Kits for DNA extraction, restriction, and ligation, oligonucleotides, and Taq polymerase were purchased from Evrogen (Moscow, Russia) and Thermo Fisher Scientific RU (Moscow, Khimki, Russia); kanamycin was produced by Sintez (Moscow, Russia). Yeast extract, bactoagar, tryptone, and peptone were purchased from “Helicon” and “Dia-M” (Moscow, Russia). DNA and protein molecular weight markers were purchased from BioRad (Hercules, CA, USA).

### 2.2. Construction of the Plasmid pET40 CamSP

The recombinant plasmid pET40 CamSP was constructed by inserting into the NcoI/XhoI region of the plasmid pET-40b(+) (Thermo Fisher Scientific-RU, Moscow, Khimki, Russia) the full-length protease-encoding gene, synthesized by polymerase chain reaction (PCR) without signal peptide, using the genomic DNA of *C. amphilecti* KMM 296 (Collection of Marine Microorganisms, PIBOC FEB RAS). The reaction mixture contained per 10 µL: 1 µL–10× Encyclo buffer, 0.2 µL–50× Encyclo polymerase mix (Encyclo PCR kit; Evrogen, Moscow, Russia), 0.2 µL–50× dNTP mix (10 mM each), forward and reverse primers (1 µL of 5 µM each), and 1 µL–20 ng DNA. The amplification process consisted of 38 PCR cycles (15 s–95 °C, 1.4 min–72 °C). After amplification, the PCR product was purified via electrophoresis in 1% agarose gel.

The PCR product (1 µg) was treated with the restriction enzymes NcoI and XhoI in an optimal buffer (Thermo Fisher Scientific RU) for 3 h at 37 °C, which were removed from the reaction mixture using a phenol treatment (1:1). To the aqueous fraction containing the PCR product, 1/10 volume of 0.3 M sodium acetate (pH 5.2) and 1/2 volume of isopropyl alcohol were added, then incubated at −20 °C for 30 min. After centrifugation at 14,000× *g* rpm for 20 min, the precipitate was washed with 75% ethanol and then dried at a room temperature. The precipitate was dissolved in 20 µL of deionized water. A total of 2 µg of the pET-40b(+) DNA (Thermo Fisher Scientific-RU) was treated with NcoI and XhoI, as described above. The resulting fragment of the CamSP gene and the NcoI/XhoI part of pET-40b(+) were ligated in 50 μL of ligation buffer according to the instructions (Novagen). Then, 10 μL of the reaction mixture was used to transform competent E. coli Rosetta (DE3) cells. The transformants were grown on Luria-Bertani (LB) agar containing 50 μg/mL kanamycin. After incubation for 16 h at 37 °C, the clones were screened, and the target plasmid DNA was isolated and sequenced.

### 2.3. Production of the Recombinant Protease CamSP

The recombinant strain of *E. coli* Rosetta (DE3) was grown in 25 mL of the liquid LB medium containing 25 mg/mL of kanamycin at 200 rpm for 16 h at 37 °C. Then, the cells were placed in a fresh LB medium (1 L) containing kanamycin at the concentration of 25 mg/mL and incubated at 37 °C on a shaker at 200 rpm until the optical density at 600 nm was 0.6–0.8. After that, 0.2 mM isopropyl-β-D-thiogalactopyranoside (IPTG) was added to induce the CamSP gene expression, and incubation was continued at 37 °C for 6 h at 200 rpm. Cells were pelleted via centrifugation at 4000× *g* rpm for 15 min at 8 °C, suspended in 35 mL of 25 mM Tris-HCl buffer (pH 7.5), and subjected to an ultrasonic treatment at a frequency of 22 kHz and 0–4 °C at intervals 30 s to clarify the suspension. The suspension was centrifuged at 11,000× *g* rpm for 30 min at 8 °C, the precipitate was discarded, and the proteolytic activity of CamSP was determined in the resulting extract (described below). Protein concentration was measured according to the Bradford method [27] using bovine serum albumin (BSA) as a reference.

### 2.4. Isolation and Purification of the Recombinant Protease CamSP

For the CamSP isolation, the resulting supernatant after ultrasonic treatment was applied to a 25 × 3.2 cm Ni-IMAC-Sepharose column (Cytiva (GE Healthcare) Life Sciences, Buckinghamshire, UK), equilibrated with 50 mM Tris-HCl, pH 7.5 (buffer A), and washed with five volumes of the same buffer. The recombinant protein was eluted with a linear gradient of 0–0.5 M imidazole in 50 mM Tris-HCl buffer, pH 7.5, and 0.5 M NaCl (6 volume of the column) at a rate of 1.3 mL min^−1^. The fraction containing CamSP was purified on a 10 × 1.4 cm Source 15 Q column (Cytiva (GE Healthcare) Life Sciences) and equilibrated by buffer A with 1 mM MgCl_2_ (buffer B). The protein was eluted with a linear gradient of 0–0.5 M NaCl in buffer B. Ion exchange chromatography was performed at 1 mL min^−1^; the volume of fractions was 1 mL. The fractions containing CamSP were collected and treated with enterokinase at a final concentration of 1 unit per 1 mg of protein for 22 h at 25 °C. After the enterokinase treatment, the protein solution was applied to a HisTrap™ High-Performance column (Cytiva (GE Healthcare) Life Sciences) and pre-equilibrated with buffer B. The recombinant protein was eluted with 10 volumes of buffer B by a linear gradient of 0–0.5 M imidazole and 0.5 M NaCl at a rate of 0.5 mL min^−1^. The fractions containing CamSP were collected and chromatographed using a mono-Q HR (4 × 0.8 cm) column (Cytiva (GE Healthcare) Life Sciences, UK), equilibrated with buffer B, and then washed with 10 column volumes of buffer B. The target protein was eluted by linear gradient of 0–0.5 M NaCl in buffer B at a rate of 0.5 mL min^−1^ as 1 mL fractions. The purified CamSP preparation was used to study the physical–chemical properties and substrate specificity.

### 2.5. Determination of the CamSP Proteolytic Activity

The protease activity was determined using the method described earlier [28], using 1% casein or bovine serum albumin (BSA) as the substrates (Sigma-Aldrich, St. Louis, MO, USA). The enzyme solution, diluted to the required concentration, was mixed with 0.5 mL of 50 mM Tris-HCl buffer, pH 7.5, containing 1% casein (or BSA) and incubated for 20 min at 50 °C. The reaction was stopped by adding cold 10% trichloroacetic acid (TCA). The acid-soluble product was determined spectrophotometrically at a wavelength of 280 nm. The standard curve was built using the solutions of tyrosine at a concentration of 0–100 µg L^−1^. One unit (U) of protease activity was defined as the amount of the enzyme that releases 1 µg of tyrosine in 1 min under the experimental conditions used. Protease activity is the average of two measurements taken in triplicate. The difference between the values did not exceed 5%.

### 2.6. Influence of pH on the CamSP Proteolytic Activity

The optimal pH was determined in a range of pH 3.2–10.0, using 1% casein as a substrate. The following buffer solutions were used: 100 mM sodium acetate (pH 3.2–6.2), 100 mM Tris-HCl (pH 6.0–10.0), glycine-NaOH (pH 8.0–10.0), and phosphate buffer (5.2–8.2).

### 2.7. Determination of the CamSP Thermal Stability and Optimum Temperature

The effect of temperature on the CamSP stability and the optimum temperature for manifestation of its proteolytic activity were determined by incubating the standard incubation mixture for the proteolytic activity measurement at 15 to 65 °C at intervals of 5 to 10 °C. The proteolytic activity was determined using the standard method described above.

### 2.8. Effect of Divalent Metal Ions and Inhibitors on the CamSP Proteolytic Activity

The effect of divalent metal ions on the CamSP proteolytic activity was evaluated using the standard method for determining the proteolytic activity by the addition of Mg^2+^, Ca^2+^, Mn^2+^, Zn^2+^, Co^2+^, Ni^2+^, Cu^2+^, and Li^2+^ to the incubation mixture at a final concentration of 2–10 mM. The effects of chelating agents (EGTA, EDTA), solvents, and detergents on the CamSP proteolytic activity were evaluated by the addition of PMSF, triton-X-100, SDS, ethanol, isopropanol, and glycerol to the incubation mixture in various concentrations. The incubation mixture without cations, chelating agents, solvents, and detergents was used as the control.

### 2.9. Effect of NaCl and KCl on the CamSP Proteolytic Activity

The salt tolerance was studied by adding NaCl and KCl to the standard incubation mixture at a concentration of 0–1.5 M. The CamSP proteolytic activity was determined as described above.

### 2.10. Determination of the CamSP Molecular Weight

The molecular weight of CamSP was determined using polyacrylamide gel electrophoresis under denaturing conditions (SDS-PAGE, polyacrylamide gel electrophoresis with sodium dodecyl sulfate) according to the Laemmli method [29] and gel filtration on a calibrated Superdex 200 PG column (105 × 2 cm) (GE Healthcare) Life Sciences, Buckinghamshire, UK).

### 2.11. Determination of the CamSP Substrate Specificity

The substrate proteolytic specificity of CamSP was determined using the synthetic chromogenic substrates α-benzoyl-Arg-p-nitroanilide (BAPNA) and N-succinyl-L-alanyl-L-alanyl-L-prolyl-L-phenylalanine 4-nitroanilide (SAPNA) (Sigma-Aldrich, St. Louis, MO, USA) and natural substrates, such as casein, bovine serum albumin (BSA), as well as corn, wheat, and soybeans, purchased at a domestic market.

The standard 25 mM solutions of BAPNA and SAPNA were prepared in 5% dimethyl sulfoxide (DMSO) immediately before the experiment. Protease activity was analyzed by mixing 0.5 mg of the purified enzyme CamSP and 1 mL of assay buffer containing one of the substrates (1 mM BAPNA or 20 mM SAPNA with 50 mM Tris-HCl, pH 7.5). After the 30 min incubation at 50 °C, the reaction was stopped by adding 1 mL of cold 10% trichloroacetic acid (TCA) and kept at a room temperature for 15 min. The increase in absorbance due to protein hydrolysis and release of p-nitroanilide were measured at the corresponding wavelength of the original substrate. One unit of activity was taken as the amount of the enzyme that released 1.0 mM p-nitroanilide per min, the concentration of which was determined using a molar absorption coefficient of 10,500 M^–1^ cm^–1^ at a wavelength of 410 nm [30].

The flour was ground from each type of grain (soybean, wheat, or corn) in a household blender; then, a 1% solution of the resulting flour was prepared in buffer A (50 mM tris-HCl, pH 7.0, 0.3 M NaCl, 10% ethanol) and incubated at 37 °C for 60 min, with constant stirring. After incubation, the solutions were centrifuged for 5 min at 10,000× *g* rpm, and the optical density in the supernatant was measured. After that, each sample was diluted with buffer A to an optical density of approximately 0.5 at 280 nm, and the protease CamSP was added. The reaction was carried out for 60 min at 50 °C and then stopped with one volume of the cold 10% TCA. The optical density was measured at 280 nm. Buffer A without the flour, but with the addition of the CamSP enzyme, was used as the control.

### 2.12. Determination of Kinetic Parameters for the Protease CamSP

The kinetic parameters of CamSP were calculated from the initial reaction rates using bovine serum albumin (BSA) as a substrate at concentrations from 10 to 100 μg mL^−1^ in 50 mM Tris-HCl, pH 7.5, at 50 °C. Each reaction was carried out for 5 min in triplicate. The Michaelis constant (*K*_m_), the maximum reaction rate (V_max_), and the turnover number (k_cat_) were determined by plotting the Lineover–Burk plot using the OriginPro 8.5 program.

### 2.13. Analysis of Nucleotide and Amino Acid Sequences

Analysis of the CamSP homology was carried out using the BLAST program available on the portal of the US National Center for Biotechnology Information (NCBI), with Nucleotide blast algorithm (https://blast.ncbi.nlm.nih.gov/Blast.cgi, accessed on 20 April 2023), MEROPS (http://merops.sanger.ac.uk/, accessed on 20 April 2023), and UNIPROT (http://uniprot.org, accessed on April, 2023). The amino acid sequence of signal peptide was determined using the SignalP-5.0 server (https://services.healthtech.dtu.dk/service.php?SignalP-5.0, accessed on 20 April 2023). The protein CamSP characteristics (molecular weight, amino acid composition, aliphatic index, GRAVY index) were obtained using the ProtParam program on the ExPASy portal (http://web.expasy.org/protparam/, accessed on 20 April 2023). Secondary structure of the protein CamSP was calculated using the SOPMA program (https://npsa-prabi.ibcp.fr/cgi-in/npsa_automat.pl?page=/NPSA/npsa_sopma.html, accessed on 20 April 2023). Tertiary structure of the protein CamSP was predicted via AlphaFold v2 with the use of 3D model of the predicted DegP-type protease from *Cobetia* sp. ICG0124 (85.654% sequence identity) as a template found by AFDB search (A0A3T0K7V4|SWISS-MODEL Repository (expasy.org), accessed on 31 May 2023). Phylogenetic analysis was carried out using MEGA 11 (Molecular Evolutionary Genetics Analysis) software package (http://www.megasoftware.net/, accessed on 20 April 2023). Ancestral states were inferred using the Maximum Likelihood method and JTT matrix-based model. The tree used a set of possible amino acids (states) at each ancestral node based on their inferred likelihood at site 1. For each node, only the most probable state was shown. Initial trees for the heuristic search were obtained by applying Neighbor-Join and BioNJ algorithms to a matrix of pairwise distances estimated using the JTT model and then selecting the topology with superior log likelihood value. The rates among sites were treated as being uniform among sites (uniform rates option). The analysis involved 16 amino acid sequences, which were taken from the NCBI and MEROPS databases according to the BLAST search results against the protease CamSP as the query sequence. There were 482 positions in the final dataset in total.

## 3. Results and Discussion

### 3.1. Structural Classification and 3D Modelling of CamSP

Based on the structural classification and sequence analysis in the MEROPS database (http://merops.sanger.ac.uk/, accessed on 20 April 2023), the protein CamSP from *C. amphilecti* KMM 296 was concluded to belong to the S1C subfamily of DegP-type periplasmic serine endoproteases of clan PA(S) [5]. Among available structures in the SWISS-MODEL repository of the MEROPS database [5,31], the AlphaFold model of *Cobetia* sp. ICG0124 (gene: A0A3T0K7V4_9GAMM, seq identity 85.65%) built itself on the base of the crystal structure of *Helicobacter pylori* HtrA (7xs0.1.A), with an average model confidence (pLDDT) equal to 76.62. The crystal structures of *H. pylori* HtrA (monomer and homo-trimer PDB ID: 7xs0.1.A, seq identity 35.80%); *E. coli* DegP (homo-12-mer PDB ID: 6jjk.1.G, seq identity 35.87%); *Legionella pneumophila* proteinase Do (homo-12-mer PDB ID: 4ynn.1.A, seq identity 35.41%), *Campylobacter jejuni* proteinase DegQ (homo-12-mer PDB ID: 6z05.1.A, seq identity 36.17%); chloroplastic protease Do-like 2 and 9 (homo-hexamer PDB ID: 5ilb.1.A, seq identity 27.22%) were chosen by AlphaFold for the construction of eight 3D models for the *C. amphilecti* KMM 296 protease CamSP (Figure S1a–c).

Due to a common HtrA/DegP/Q catalytic triad of His-Asp-Ser and two PDZ domains, which mediate substrate recognition and oligomerization [9,13,14,16], the *C. amphilecti* KMM 296 protease CamSP has 36.15% identity with the high-temperature requirement human mitochondrial trimeric protease HtrA2 (Protein Data Bank (PDB) ID: 5m3n.1, chain A). The human HtrA2 is in a list of the top templates for 3D modelling CamSP, according to the Swiss-Prot analysis [5] (Table S1). The oligomerization up to 24-mers for the HtrA/DegP/Q-type enzymes is thought to be an allosteric regulation to switch the active and inactive conformations. Allosteric effects may be observed between the individual domains of monomeric proteins DegP or between the protomers of the oligomeric protein complexes [10].

### 3.2. Calculated Structural Properties and Phylogenetic Relatedness of CamSP

The *C. amphilecti* KMM 296 mature protease CamSP consists of 666 aa residues and has a putative signal peptide of 25 aa residues, identified using the SignalP-5.0 server (https://services.healthtech.dtu.dk/service.php?SignalP-5.0, accessed on 20 April 2023) [32]. Analysis of the aa sequence of the *C***.** *amphilecti* protease CamSP by the SOPMA server (NPS@: SOPMA secondary structure prediction (ibcp.fr), accessed on 20 April 2023) revealed four classes of secondary structures, namely: random turns, α-helices, extended strands, and β layers in the percentages of 36.49, 27.33, 24.92, and 11.26, respectively [33]. The GRAVY index of CamSP, analyzed by the ProtParam server and equal to −0.097, indicates that the protease has hydrophilic properties and is highly soluble in aqueous buffer solutions [33].

**Figure 1:**
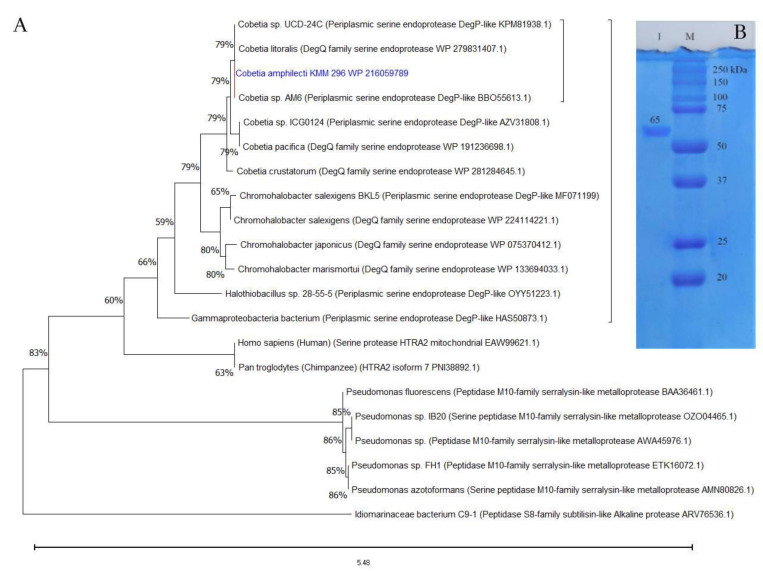
The protein structure analysis and molecular weight of the *C. amphilecti* KMM 296 protease CamSP: (**A**) Phylogeny reconstruction for protease CamSP and closest relatives using the Maximum Likelihood method and JTT matrix-based model (MEGA11, http://www.megasoftware.net/, accessed on 20 April 2023); (**B**) SDS-PAGE of the purified recombinant protein CamSP (line 1); M—molecular weight marker (BioRad).

According to the BLAST-based search results in MEROPS and NCBI databases, the aa sequences of CamSP-like proteins from the species of *Cobetia* are much closer to the biochemically studied highly halothermotolerant proteases from *Chromohalobacter* sp. TVSP101 [34] and *Chromohalobacter salexigens* BKL5 [35], with an identity of approximately 62%. The phylogenetic tree reconstruction allowed for clustering the CamSP-like proteins of *Cobetia* spp. separately from other S1C subfamily proteins, indicating a new structural member of the DegP/Q-type serine protease family (Figure 1A).

### 3.3. Heterologous Expression and Isolation of CamSP

Heterologous expression of the *C. amphilecti* KMM 296 gene (GenBank ID: WP_216059789.1) corresponding to the mature protein CamSP in the *E. coli* Rosetta DE(+) cells resulted in the production of a soluble recombinant protein CamSP, with a proteolytic activity of 1550.9 U/mg towards the casein substrate (1%) after its purification, according to the scheme described in Table 1.

The isolation of enzymatically active recombinant protein CamSP confirmed the *C. amphilecti* ability to produce the functionally active DegP-type protease, with a calculated molecular weight of 69,957 kDa for the mature protein, which is consistent with the estimation of its molecular weight using polyacrylamide gel electrophoresis (PAGE) and gel filtration on Superdex-200 (Figure 1B). According to the results of SDS-PAGE, the molecular weight of CamSP is 65 ± 5 kDa (Figure 1), which coincides with the *Chromohalobacter* sp. TVSP101 protease of 66 kDa [34], but it is higher than the weight of DegP protease of 45 kDa isolated from *C. salexigens* BKL5 [35] that has been found to form the active multimers of 297.9 and 579.12 kDa [35].

### 3.4. Effect of pH on CamSP Activity

**Figure 2:**
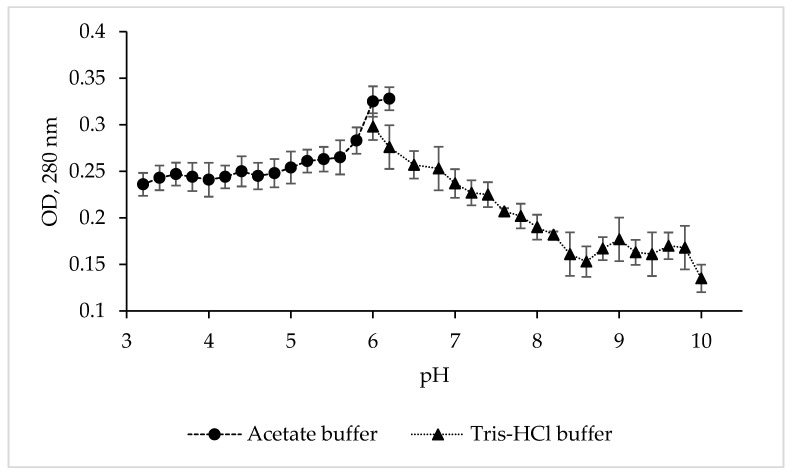
Effect of pH and buffers on the proteolytic activity of *C. amphilecti* KMM 296 protease CamSP with the use of 1% casein as substrate.

To determine the pH optimum and stability of the protease CamSP, the enzyme activity was studied in various buffer solutions over a wide pH range (Figure 2).

Although CamSP is stable from pH 5.8 to 8.5, its activity was at the highest level at pH 6.0–6.2 in 0.1 M acetate buffer (Figure 2), similar to the serine proteases from *Thermoactinomyces* sp., *Alkalihalobacillus lehensis* JO-26, and *Pseudoaltermonas* sp. SM9913 (Table 2). The glycine-NaOH (pH 8.0–10.0) and phosphate (5.2–8.2) buffers strongly decreased the activity of CamSP. The other most characterized bacterial proteases have much higher levels of pH optimum at 8.0 to 11.0, especially thermo- and salt-tolerant enzymes (Table 2). However, DegP-like proteases have been reported to be a virulence factor in pathogenic bacteria, which are often endocytosed into the lysosomes, an acidic subcellular compartment, of the host cells [11]. Moreover, enteric bacteria like pathogenic *E. coli* have evolved an acid stress resistance mechanism involving the DegP protease function for surviving in the host stomach conditions. Although the *E. coli* DegP protease activity is completely lost under acidic conditions, it is substantially restored after neutralization at around pH 5.5 and participates in the degradation of acid-denatured periplasmic proteins toxic to the cells [11,12].

### 3.5. Effect of Ionic Strength on CamSP Activity

**Figure 3:**
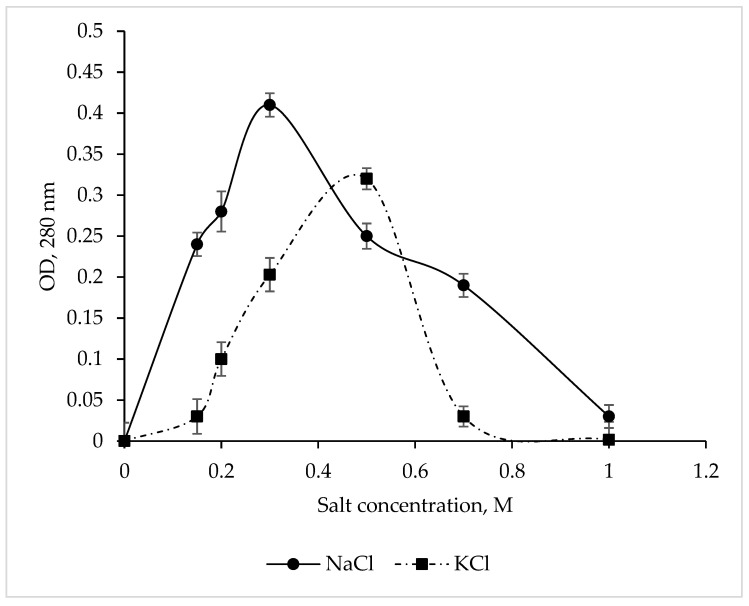
Effect of NaCl and KCl on the *C. amphilecti* KMM 296 protease CamSP activity.

The CamSP protease exhibited maximum activity at 0.3 M NaCl and 0.5 M KCl (Figure 3), while Na^+^ and K^+^ salts at a concentration of 1 M completely inhibited the enzyme. These results are in complete agreement with the data obtained for CamSP in silico using the ProtParam program (http://web.expasy.org/protparam/, accessed on 20 April 2023) [44]. The *C. amphilecti* KMM 296 alkaline phosphatases CmAP and CmPhoD have the same moderate salt dependence, indicating their extracellular functions and the marine environment habitat of their producing microorganism [45,46].

The in silico analysis of the aa residues in CamSP showed that the protein composition is dominated by acidic amino acids, while the ratio of alkaline and acidic amino acids is 0.9. Comparing the obtained ratio with nonhalophilic and extremely halophilic archeons [35], the experimental data on the CamSP protease confirmed that it is a nonhalophilic protein.

### 3.6. Effect of Divalent Metals on CamSP Activity

The various salts of Zn^2+^, Cu^2+^, Mn^2+^, Li^2+^, Mg^2+^, and Ca^2+^ slightly activated CamSP, while adding CoCl_2_ to the incubation mixture resulted in a twofold increase in the protease activity (Table 3), similar to the Co^2+^ dependence of the *C. amphilecti* KMM 296 alkaline phosphatase CmPhoD [46]. In addition, the marine microorganism *C. amphilecti* KMM 296 was found to possess a lot of magnesium and cobalt transporters, efflux proteins (CorC, A), and cobalt–zinc–cadmium resistance proteins (CzcD), suggesting significant environmental exposure to these metals and an involvement of the mollusk-associated strain KMM 296 in bioremediation and redox cycling of the transition metals [47,48,49].

### 3.7. Effect of Detergents, Chelators, and Organic Solvents on CamSP Activity

The effects of anionic (SDS) and nonionic (Triton X-100) surfactants, the inhibitors for serine proteases (PMSFs) and metalloproteases (EDTA and EGTA), organic solvents (ethanol and isopropanol), and glycerol on the *C. amphilecti* KMM 296 protease CamSP are presented in Table 4. The protease CamSP was slightly inhibited by the chelating agents and 50% by SDS (Table 4). With increasing the EDTA concentration, the activity of CamSP decreased, confirming the metal ions’ influence on the level of protease activity. The enzyme expectedly exhibited reduced activity in the presence of the serine protease inhibitor PMSF, suggesting that the CamSP protease belongs to the class of serine proteases (Table 4). Ethanol and isopropanol increased the activity of CamSP by an average of 4-times and glycerol and Triton-X-100 by 2-times, which are consistent with the data on some proteases, for example, a *Serratia marcescens* metalloprotease [25], making the *C. amphilecti* protease useful for industrial applications such as peptide synthesis [39,50].

### 3.8. Effect of Temperature on CamSP Activity

**Figure 4:**
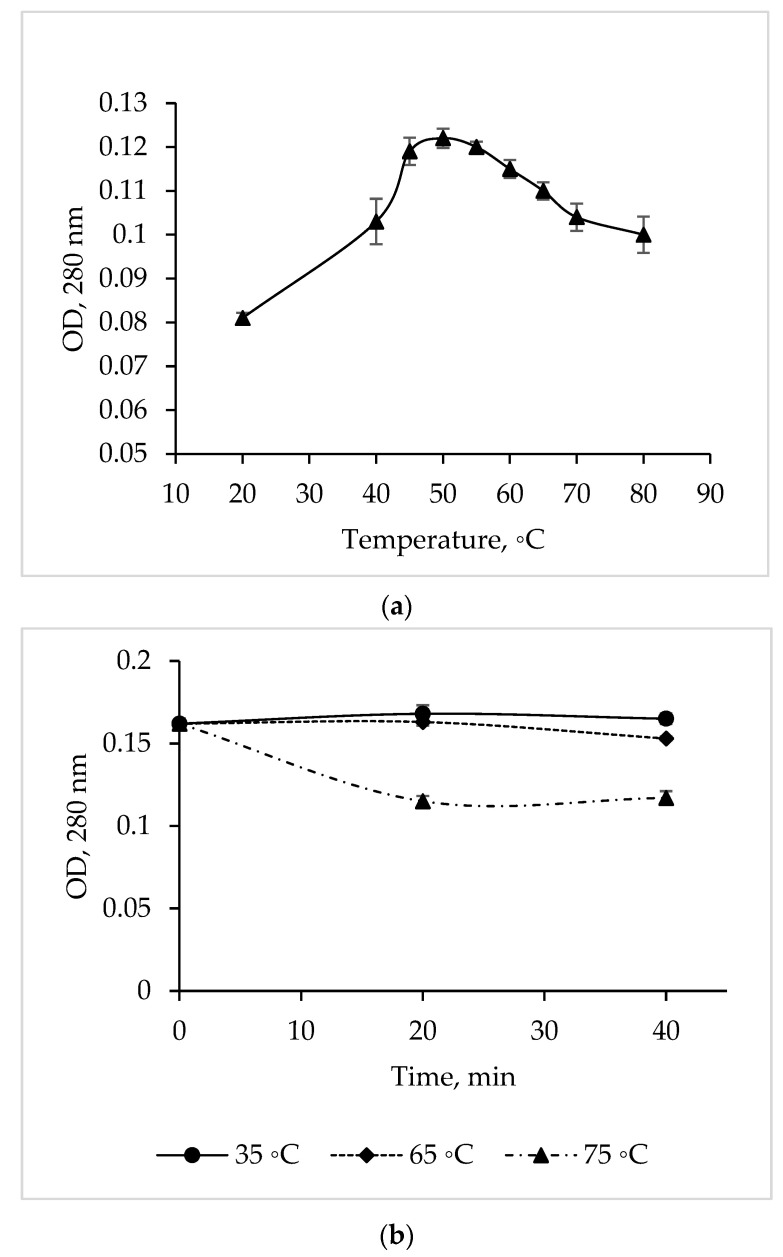
Temperature optimum (**a**) and thermal stability (**b**) of the *C. amphilecti* KMM 296 protease CamSP.

The temperature dependence study showed that the maximum activity of the *C. amphilecti* KMM 296 protease is at 50 °C, but the enzyme activity remained at a high level in a temperature range of 45–55 °C (Figure 4a).

The CamSP proteolytic activity was 100% from 35 to 65 °C for 40 min (Figure 4b). In addition, the activity of CamSP decreased by 27% after 20 min of its pre-incubation at 75 °C (before the proteolysis reaction); then, the enzyme retained 73% of the initial activity for another 20 min (Figure 4b). The long-time thermostability of CamSP is in accordance with the known biological functions of the DegP-like proteases, being essential for bacterial growth under high-temperature conditions [11]. According to Figure 4 and the aliphatic index of 96.25 obtained by analyzing the CamSP aa sequence with the ProtParam program (http://web.expasy.org/protparam/, accessed on 20 April 2023), the *C. amphilecti* KMM 296 protease is a moderately thermostable enzyme, similar to the serine metalloproteases from the marine host- and sediment-associated *Bacillus subtilis* GA CAS8 and *Pseudoaltermonas* sp. SM9913 MPC-02, respectively [22,26].

### 3.9. Substrate Specificity of CamSP

**Figure 5:**
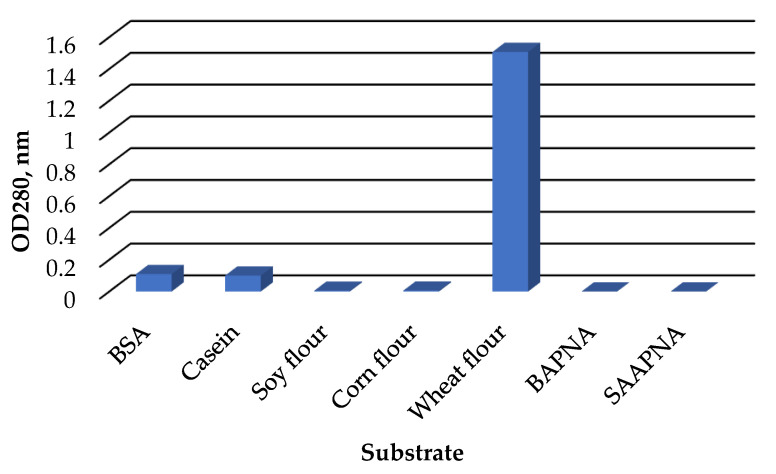
Relative proteolytic activity of *C. amphilecti* KMM 296 protease CamSP towards proteins of different origin.

The *C. amphilecti* KMM 296 protease CamSP exhibited the proteolytic activity towards natural substrates, such as casein, bovine serum albumin, and wheat flour proteins (Figure 5). However, CamSP did not degrade soybean, corn, and synthetic substrates α-benzoyl-Arg-*p*-nitroanilide (BAPNA), N-Succinyl-L-alanyl-L-alanyl-L-prolyl-L-phenylalanine 4-nitroanilide (SAPNA) (Figure 5).

The widely used substrate for the different studies on bacterial serine proteases with various proteolytic activities was casein (Table 2), although the *E. coli* DegP-type protease activity towards this substrate and several other nonnative substrates was weak [12]. The natural DegP targets were identified as the colicin A lysis protein, bacterial pilins, nonpilus adhesins, and partially unfolded proteins to gain access to their catalytic sites for cleaving by DegP protease between paired hydrophobic residues [12]. Generally, HtrA/DegP/Q-type proteases cleave peptide bonds preferentially between such hydrophobic residues as Val-Val or Ile-Xaa [9,13,14,16].

**Figure 6:**
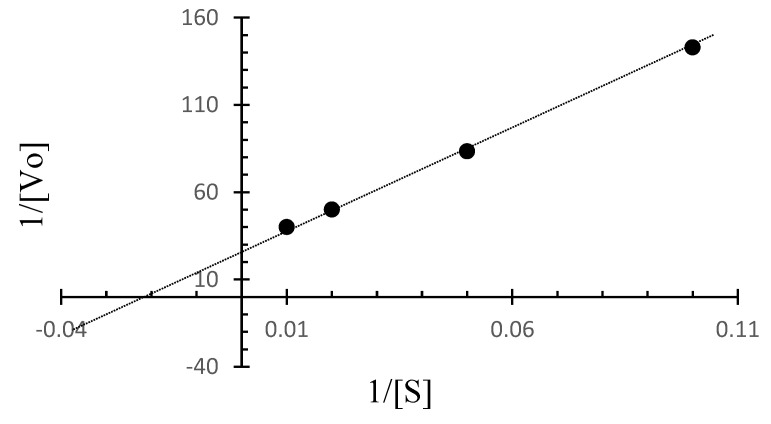
The Lineweaver-Burk plot for CamSP based on the initial rates obtained for BSA at different concentrations at 50 °C.

The Lineweaver–Burk plot for *C. amphilecti* KMM 296 protease CamSP was built based on the initial rates for BSA as the substrate at different concentrations and at a temperature of 50 °C (Figure 6). Based on the Lineweaver–Burk plot, the kinetic parameters for CamSP are V_max_ = 0.036 µg/mL min^−1^ and *K*_m_ = 41.7 µg/mL, 1/2 V_max_ = 0.018 µg/mL min^−1^, k_cat_ = 529.42 min^−1^, and k_cat_/*K*_m_ = 14.706 min^−1^.

The substrate specificity and catalytic properties of the enzyme CamSP may be applicable in the pharmaceutical, food, and bakery industries for the hydrolysis of wheat gluten and the production of gluten-free products for medical nutrition [17].

## 4. Conclusions

The gene of the marine bacterium *C. amphilecti* KMM 296 under the GenBank ID: WP_216059789.1, expressed in *E. coli* cells, was confirmed to encode for the metabolically active protease belonging to the structurally new periplasmic serine endoprotease/chaperon of the DegP type of clan PA(S). The *C. amphilecti* KMM 296 protease CamSP is a moderately thermostable, metal-dependent, and solvent-tolerant proteolytic enzyme, which is in a monomeric state under the used conditions (50 mM Tris-HCl buffer, pH 7.5) and preferentially cleaves the proteins of milk, wheat, and blood serum at neutral pH, suggesting its food and pharmaceutical use potential.

## Figures and Tables

**Table 1:** Purification scheme for the recombinant *C. amphilecti* KMM 296 protease CamSP.

Purification Step	Total Proteolytic Activity (Units)	Total Protein (mg)	Proteolytic Activity (U mg^−1^)	Yield (%)	Purification (Fold)
Crude homogenate	431 × 10^3^	650.8	585.3	100	1
Ni-IMAC-Sepharose	111.6 × 10^3^	108.3	1030.6	26	1.7
Source 15 Q	15.9 × 10^3^	19.3	823.1	4	1.4
HisTrap™ HP	12.4 × 10^3^	8	1550.9	3	2.6

**Table 2:** Comparative physical–chemical and catalytic characteristics of microbial proteases.

Strain	Classification	Proteolytic Activity, U/mg	Optimum pH	Optimum T °C	NaCl, M	Weight, kDa	Metal Ions	*K*_m_	Substrate	Resistance	References
*Cobetia amphilecti* KMM 296 (WP_216059789)	Serine protease	1550.9	6.0-6.2	50	0.3	65	Co^2+^	41.7 µg/mL min^−1^	Casein, BSA	Ethanol, Isopropanol	This study
*E. coli* (NP_414703)	DegP protease	-	7.4-8.0	45	-	47	Mg^2+^ Mn^2+^ Ca^2+^	-	β-Casein	-	[11,12]
*Chromohalobacter* sp. TVSP101	Extremely Halophilic Thermophilic protease	5225	8.0	75	4.5	66	Mg^2+^ Ca^2+^	-	Azocasein	DMSO DMF Ethanol Acetone	[34]
*Chromohalobacter salexigens BKL5* (MF071199)	Serine protease	-	-	30	-	45	-	-	Casein	-	[35]
*Pseudoaltermonas* sp. SM9913 MPC-01 (AY305857)	Serine protease	2890.1	6.5–7.0	30–35	-	60.7	Ca^2+^	0.18%	Casein	-	[26]
*Pseudoaltermonas* sp. SM9913 MPC-02	Mesophilic metalloprotease	536.3	8.0	50–55	-	36	Zn^2+^	0,36%	Casein	-	[26]
*Pseudomonas* sp. AU10 (MF375895)	Cold-active serine-metalloprotease	109.7	8.0	40	-	50	Ca^2+^	-	Azocasein	-	[36]
*Pseudoalteromonas* sp. CP76	serine metalloprotease	133	8.5	55	1	38	-	7.1 mκM	Casein	Bestatin, Himostatin, Leipeptin, Pepstatin	[37]
*Bacillus licheniformis RP1*	Serine thermophilic protease	233	10.0–11.0	65–70	-	27.5	Ca^2+^	-	Casein	Tween 20% Triton X-100, SDS	[21]
*Bacillus subtilis* GA CAS8 (JX627400)	Salt tolerant metalloprotease	87.79	9.0	50	-	41	Mg^2+^ Ca^2+^	-	Peanut cake, cabbage leaf	Tween 20, Tween 40 and SDS.	[22]
*Serratia marcescens* PPB-26 (KJ735909)	Metalloprotease	17.5	7.5	30	0.9	-	Fe^2+^ Cu^2+^	0.3%	Casein	Methanol Ethanol	[25]
*Bacillus luteus* H11	Serine halotolerant subtilisin-like endoprotease	115.2	10.5	45	3.0	37	Mg^2+^ Ca^2+^ Ba^2+^	-	Azocasein, N-succinyl-l-phenylalanine-p-nitroanilide	Tween 80 DMSO Ethanol	[38]
*Alkalihalobacillus lehensis* JO-26 (MH104891)	Serine subtilisin protease	4912	7.0	10	-	28.34	Ca^2+^	1.38 mg/mL	Casein	SDS, Chloroform, Toluene, n-Butanol Benzene	[39]
*Thermoactinomyces* sp. 27a (AY280367)	Serine thermolysin-like metalloprotease-	155	6.5–7.5	55	-	34.19	Ca^2+^	-	Azocasein, 3-(2-furyl)acryloyl-glycyl-L-leucine amide (FAGLA)	-	[40]
*Aspergillus flavus* (MT380801)	serine protease	39.035	10.0	45	-	42.57	Ca^2+^	-	Azocasein	Sucrose Tween 20 Sorbitol Glycerin H_2_O_2_	[41]
*Pediococcus acidilactici* NCDC 252	serine protease	23.3	8.5	37	-	37.1	-	38 mκM	Nα-Benzoyl-DL-arginine 4-nitroanilide hydrochloride (BAPNA)	-	[42]
*Idiomarina* sp. C9-1	serine alkaline protease	42567.1	10.5	60	-	56	Ca^2+^ Cu^2+^ Mr^2+^ Co^2+^ Mn^2+^ Ba^2+^	3.76 mg mL^−1^	Casein	H_2_O_2_, Triton X-155	[43]

**Table 3:** Influence of metal salts on the CamSP proteolytic activity.

Metal Salt	Concentration, mM	Relative Activity, %
None	-	100
ZnSO_4_	2 mM	131
CuSO_4_	2 mM	115
MnCl_2_	2 mM	116
NiCl_2_	2 mM	89
CoCl_2_	2 mM	222
LiCl_2_	2 mM	118
MgCl_2_	2 mM	136
CaCl_2_	2 mM	119

**Table 4:** Influence of different detergents, chelators, and organic solvents on the CamSP proteolytic activity.

Reagents	Concentration	Relative Activity, %
None	-	100
PMSF	5 mM	65
EGTA	50 mM	87
EDTA	5 mM	95
EDTA	50 mM	74
SDS	1%	50
Triton-X-100	1%	198
Ethanol	10%	444
Isopropanol	10%	378
Glycerol	10%	198

## Data Availability

The datasets presented in this study can be found in online repositories. The names of the repository/repositories and accession number(s) can be found in the article/Supplementary Material.

## Supplementary Materials

The following supporting information can be downloaded at: https://www.mdpi.com/article/10.3390/microorganisms11071852/s1, Table S1: Template search results against the query amino acid sequence of *C. amphilecti* KMM 296 protease CamSP obtained by the automated protein structure homology-modelling server SWISS-MODEL; Figure S1. (a): The homology modelling of the *C. amphilecti* KMM 296 protease CamSP monomer build with the use of AlphaFold DB model of A0A3T0K7V4_9GAMM (gene: A0A3T0K7V4_9GAMM, organism: *Cobetia* sp ICG0124); (b): The homology modelling of the *C. amphilecti* KMM 296 protease CamSP build with the use of the crystal structure of *H. pylori* proteinase HtrA homo-trimer (PDB ID: 7xs0.1.A); (c): The homology modelling of the *C. amphilecti* KMM 296 protease CamSP build with the use of the crystal structure of *L. pneumophila* proteinase Do homo-12-mer (PDB ID: 4ynn.1.A).
