# Supplementary figures and images for: Purification and Characterization of a DegP-Type Protease from the Marine Bacterium Cobetia amphilecti KMM 296
Source: Microorganisms. 2023 Jul 21;11(7):1852. doi: 10.3390/microorganisms11071852 (PMC10383082; doi:10.3390/microorganisms11071852)

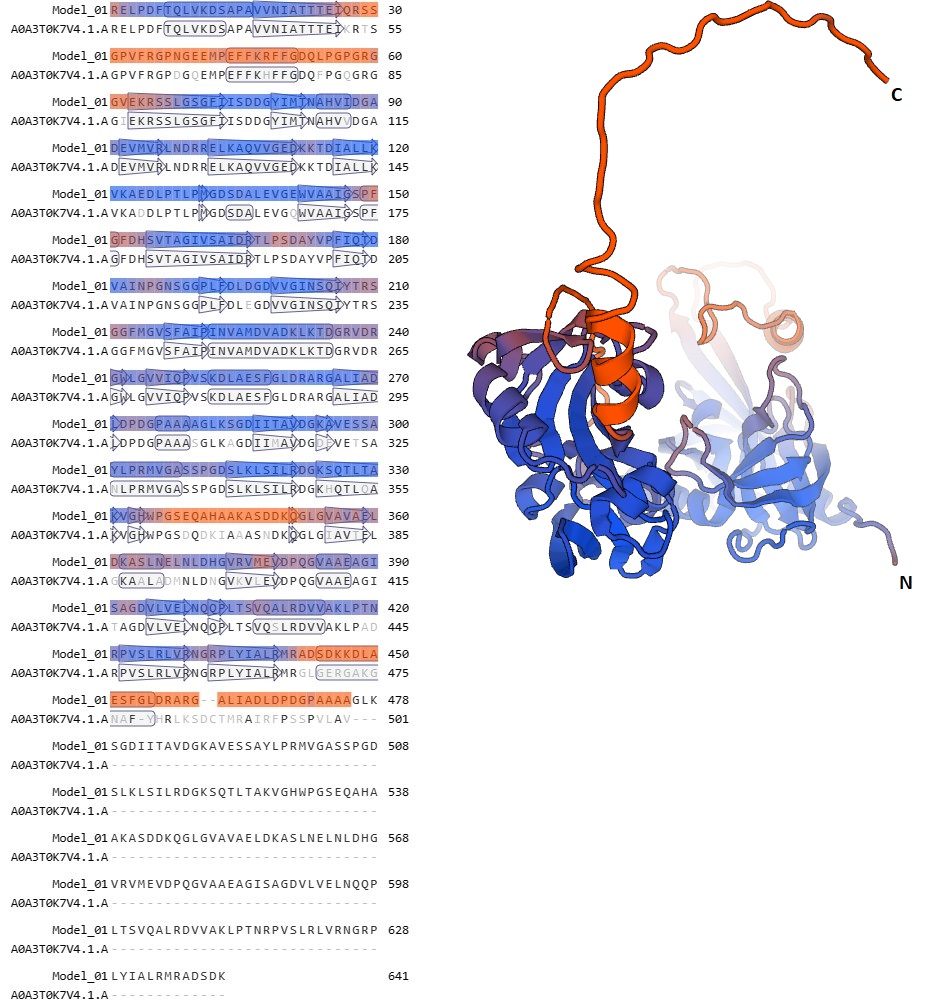

Supplement: Supplementary file 1 [file microorganisms-11-01852-s001.zip › Supplementary Figure S1 a_Monomer_Cobetia_AlphaFold model.jpg]

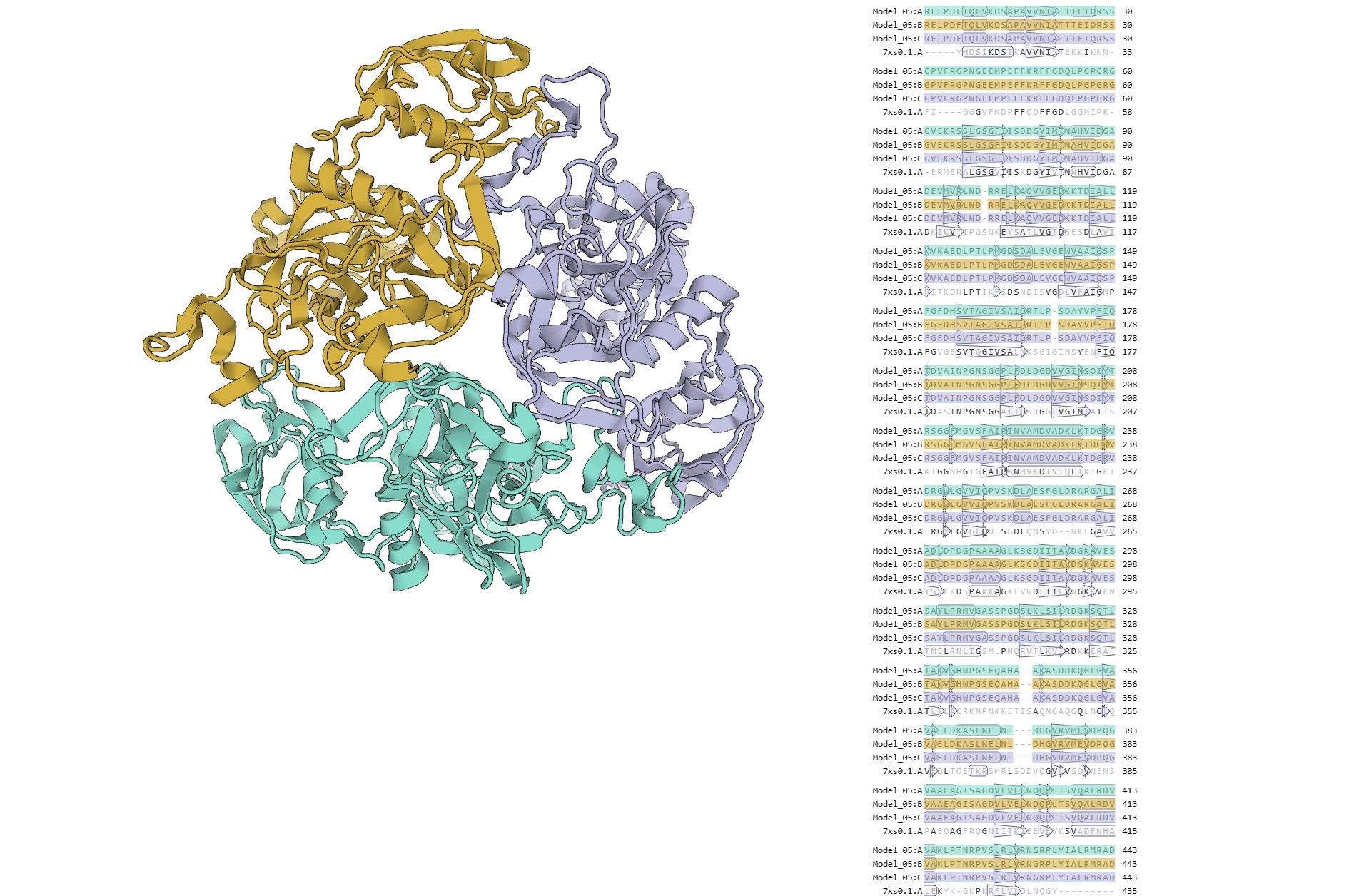

Supplement: Supplementary file 1 [file microorganisms-11-01852-s001.zip › Supplementary Figure S1 b_Homo_trimer_based on H.pylori.jpg]

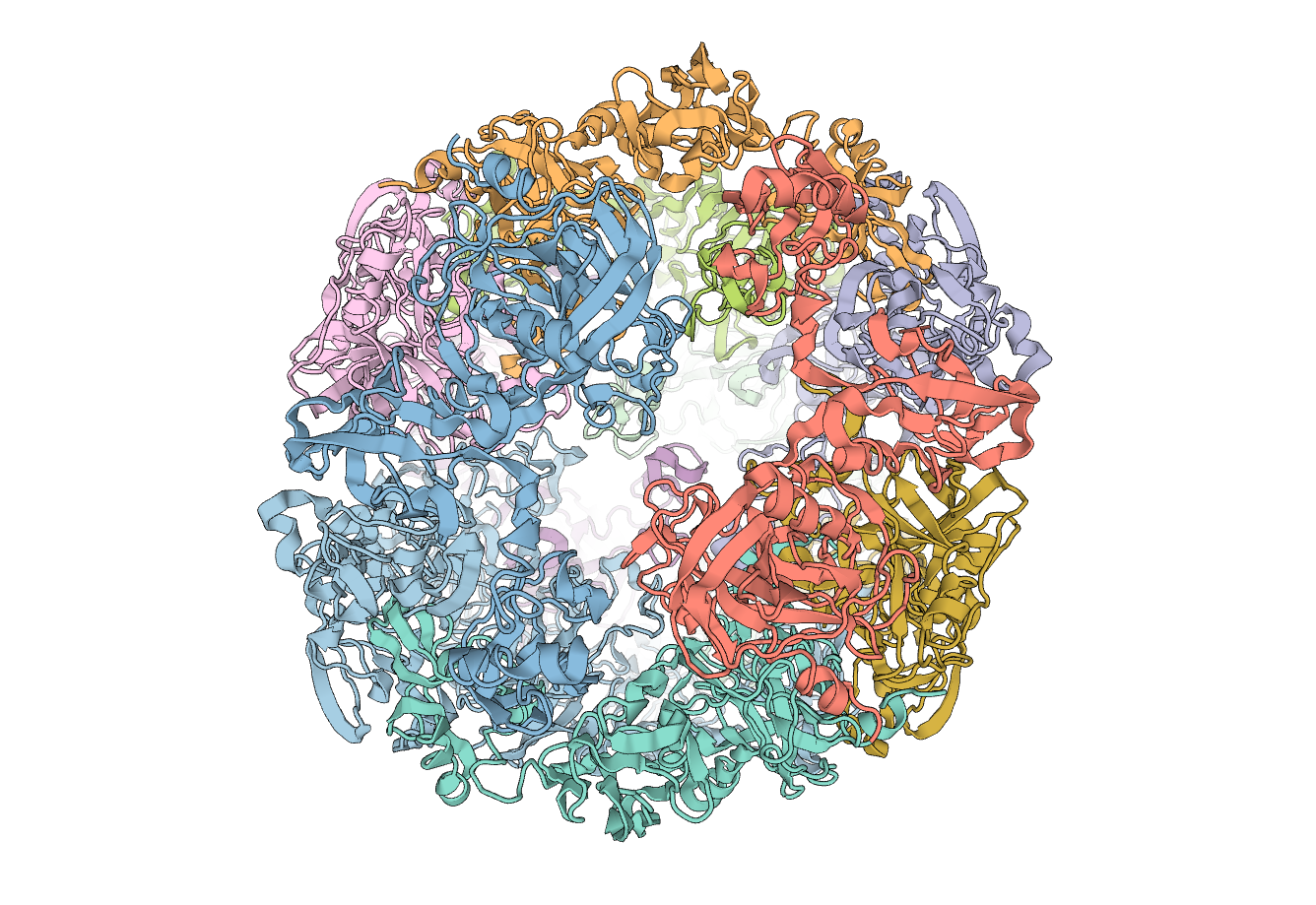

Supplement: Supplementary file 1 [file microorganisms-11-01852-s001.zip › Supplementary Figure S1 c_12mer_based on L.pneumophila DegQ.png]
